# Optimizing therapeutic approaches for HR+/HER2- advanced breast cancer: clinical perspectives on biomarkers and treatment strategies post-CDK4/6 inhibitor progression

**DOI:** 10.20517/cdr.2024.169

**Published:** 2025-01-22

**Authors:** Juan Miguel Cejalvo Andújar, Francisco Ayala de la Peña, Mireia Margeli Vila, Javier Pascual, Pablo Tolosa, Cristina Pages, Mónica Cuenca, Ángel Guerrero Zotano

**Affiliations:** ^1^Medical Oncology Department, Hospital Clínico Universitario de Valencia, Valencia 46010, Spain.; ^2^INCLIVA Biomedical Research Institute, Valencia 46010, Spain.; ^3^Center for Biomedical Network Research on Cancer (CIBERONC), Madrid 28019, Spain.; ^4^Medical Oncology Department, Hospital General Universitario Morales Meseguer, Murcia 30008, Spain.; ^5^Medical Oncology Department, Instituto Catalán de Oncología, Badalona 08916, Spain.; ^6^CARE, the Translational Program in Cancer Research of Germans Trias i Pujol Research Institute (IGTP), Badalona 08916, Spain.; ^7^Medical Oncology Department, UGC Intercentros de Oncología Médica, Hospitales Universitarios Regional y Virgen de la Victoria, IBIMA, Málaga 29010, Spain.; ^8^Medical Oncology Department, Hospital Universitario 12 de octubre, Madrid 28041, Spain.; ^9^Medical Department, Pfizer Oncology, Madrid 28108, Spain.; ^10^Medical Oncology Department, Instituto Valenciano de Oncología, Valencia 46009, Spain.

**Keywords:** HR+/HER2-, CDK4/6 inhibitors, advanced breast cancer, biomarkers, prognosis

## Abstract

This review offers an expert perspective on biomarkers, CDK4/6 inhibitor efficacy, and therapeutic approaches for managing hormone receptor-positive (HR+), human epidermal growth factor receptor-negative (HER2-) advanced breast cancer (ABC), particularly after CDK4/6 inhibitor progression. Key trials have demonstrated that combining CDK4/6 inhibitors with endocrine therapy (ET) significantly improves progression-free survival (PFS), with median durations ranging from 14.8 to 26.7 months, and overall survival (OS), with median durations reaching up to 53.7 months. Actionable biomarkers, such as *PIK3CA* and *ESR1* mutations, have emerged as pivotal tools to guide second-line treatment decisions, enabling the use of targeted therapies like alpelisib and elacestrant and emphasizing the important role of biomarkers in guiding the selection of therapy. This overview aims to provide clinicians with a practical and up-to-date framework to inform treatment decisions and improve patient care in the context of this challenging disease. Additionally, we review emerging biomarkers and novel treatment strategies to address this difficult clinical landscape.

## INTRODUCTION

In recent years, cyclin-dependent kinase 4 and 6 inhibitors (CDK4/6i), including palbociclib, ribociclib, and abemaciclib, have been approved for the treatment of hormone receptor-positive (HR+), human epidermal growth factor receptor-negative (HER2-) advanced breast cancer (ABC)^[1]^. These approvals were based on results from several phase III clinical trials (PALOMA 2 and 3, MONALEESA 2, 3, and 7, and MONARCH 2 and 3), which demonstrated that combining CDK4/6i with endocrine therapy (ET) significantly improves progression-free survival (PFS) compared to ET alone and may delay the need for subsequent chemotherapy (CT)^[2-9]^. Furthermore, follow-up analyses confirmed that combining CDK4/6i and ET provides an overall survival (OS) advantage, with reported median OS (mOS) values ranging from 34.9 months with palbociclib and fulvestrant in the PALOMA-3 trial to 53.7 months with ribociclib and fulvestrant in the MONALEESA-3 trial and 46.7 months with abemaciclib and fulvestrant in the MONARCH 2 trial^[5,8,10-13]^. However, the efficacy of these inhibitors varies among patients. Some tumors exhibit intrinsic resistance and progress rapidly, while others develop acquired resistance with prolonged use. Despite accumulating knowledge on the factors contributing to CDK4/6i resistance, this information has yet to be widely translated into clinical practice. Consequently, patients are often treated without considering this valuable biological information that could guide therapy^[14]^.

CDK4/6i (palbociclib, ribociclib, and abemaciclib) have become a cornerstone in the management of HR+/HER2- ABC. The combination of a CDK4/6i with ET is the standard-of-care first-line therapy for patients with HR+/HER2- ABC^[15]^ based on evidence from pivotal phase III trials, which demonstrated significant improvements in PFS and OS. This approach delays the need for CT and maintains patients’ quality of life (QoL), offering a significant advancement in therapeutic options. Despite these benefits, variability in treatment response and the development of acquired resistance remain significant challenges, highlighting the importance of integrating biomarkers and personalized approaches to optimize clinical outcomes.

This review aims to analyze the prognostic and predictive roles of clinical and tumor biomarkers in HR+/HER2- ABC, examining the most relevant evidence and clinical applications. We propose treatment algorithms for patients resistant to CDK4/6i and provide an overview of emerging biomarkers.

## METHODS

To provide an up-to-date overview of therapeutic approaches for HR+/HER2- ABC, we conducted a comprehensive literature search in PubMed and Scopus databases. The search primarily focused on studies published between 2016 and 2024 to ensure the review reflects the most current evidence and advancements. Earlier publications (e.g., 2002 and 2003) were also included when they provided foundational insights or remained relevant to specific aspects of HR+/HER2- advanced breast cancer (ABC) and its management. Search terms included combinations of keywords such as “HR+/HER2- ABC”, “molecular subtypes” (e.g., luminal A, luminal B, HER2-enriched), “CDK4/6 inhibitors”, “biomarkers” (e.g., *PIK3CA*, *ESR1*, *BRCA1/2*), “targeted therapies”, “endocrine therapy (ET)”, “cancer drug resistance”, “mechanisms of resistance”, “endocrine resistance”, “prognosis”, “clinical outcomes”, “progression-free survival (PFS)”, or “overall survival (OS)”, among others. Articles written in English that addressed mechanisms of drug resistance in cancer and therapeutic strategies to overcome resistance were included. Study selection was based on alignment with the objectives of this review and the methodological quality of the studies.

## MOLECULAR BREAST CANCER SUBTYPES

The histopathological classification of HR+/HER2- breast cancer (BC) does not reflect its biological heterogeneity. The classification based on specific gene expression profiles identified four main intrinsic BC subtypes, which are biologically distinct: luminal A, luminal B, HER2-enriched (HER2E), and basal-like^[16]^. Each of these intrinsic molecular subtypes can be identified in each classical pathology-based classification, albeit with different proportions. In fact, this surrogate classification has limited ability to distinguish between PAM50 luminal A and B^[17]^. Moreover, this molecular classification of BC has prognostic and predictive implications beyond classical classification. Cumulative evidence highlights the clinical value of the two non-luminal subtypes (HER2E and basal-like) in HR+/HER2- disease as representative of hormone-resistant disease^[18]^.

In the last few years, several phase III clinical trials (EGF30008, BOLERO-2, PALOMA-2, PALOMA-3, MONALEESA-2, MONALEESA-3, MONALEESA-7 and PEARL) have analyzed the molecular profile of HR+/HER2- ABC. These studies have demonstrated that the majority of tumors were luminal A and luminal B (62%-85%), both of which were associated with better prognosis. However, HER2E and basal-like tumors were also detected^[19-22]^, showing poorer response to treatment with CDK4/6i with worse PFS and OS outcomes^[22-25]^.

Intrinsic subtypes can shift between primary tumors and metastases. Thus, the basal-like subtype maintains 100% concordance, while HER2E and luminal B maintain 76.9% and 70% concordance, respectively. Interestingly, in the metastatic setting, only 44.7% of luminal A cases maintained their subtype, with 40.4% switching to luminal B and 14.9% to the HER2E subtype^[20]^. Accordingly, the PEARL study^[24]^ revealed a higher presence of non-luminal subtypes in metastatic biopsies (14%) compared to primary samples (4%), which was confirmed by data from the AURORA project^[26]^. This underscores the importance of considering the origin of the tumor sample when evaluating the prognostic and predictive value of intrinsic molecular subtypes in BC.

As discussed below, various clinical and molecular biomarkers have been identified as valuable prognostic and predictive markers for luminal ABC [Table 1].

**Table 1:** Main prognostic and predictive biomarkers in luminal breast cancer

	**Biomarker**	**Prevalence**	**Prognostic/predictive value**	**Targeted therapy**	**References**
**ACTIONABLE BIOMARKERS**	*PIK3CA* (mutated)	Primary tumor: 45% Metastases: 53%	Poor prognostic biomarker Predictive biomarker of response to specific PI3KCAi Predictive biomarker of resistance to fulvestrant Early change in mutated copies of *PIK3CA* after 15 days of treatment predict worse clinical outcomes with CDK4/6i (palbociclib)	PI3Ki (alpelisib) PI3Ki (inavolisib) + CDK4/6i (palbociclib) + ET (INAVO-120 trial; FDA-approved for first-line treatment)	[27-38]
	*ESR1* (mutated)	Primary tumor: 3.5% Metastases: 20%-48% Up to 50% after 1 year of AI + CDK4/6i treatment	Poor prognostic biomarker Predictive biomarker of response to CDK4/6i + fulvestrant after AI progression Predictive biomarker of resistance to AI + CDK4/6i (accelerated onset of AI-palbociclib resistance) Predictive biomarker of resistance to SERD (fulvestrant) - *ESR1* Y537S	New SERDs (elacestrant, and under research: camizestrant, giredestrant, amcenestrant) PROTAC estrogen receptor degrader (ARV-471) Fulvestrant + CDK4/6i (palbociclib) after AI progression	[29,39-52]
	*HER2* (mutated)	Primary tumor: 2% (mutated) Metastases: 3%-5% (mutated); 5%-8% (enriched) Lobular BC: 15% (enriched)	Prognostic biomarker Predictive biomarker of response to neoadjuvant CT, ET, anti-HER2 therapy and CDK4/6i	For mutant HER2/3 ABC (neratinib + fulvestrant + trastuzumab) Anti-HER2 (TKi: neratinib)	[53,54]
	HER2-low	Primary tumor: 34% Metastases: 37%	Predictive biomarker of response to ADC	ADC (T-DXd, SG)	[55-59]
	*BRCA1/2* (mutated)	Primary tumor: 5% (germline mutation) Metastases: 5% (germline mutation)	Germline mutations are predictive biomarkers of response to PARPi	PARPi (talazoparib, olaparib)	[60-64]
**NO ACTIONABLE BIOMARKERS**	RB1 (loss of function)	Primary tumor: 0%-3% Metastases: 11,5% (after CDK4/6i)	Predictive biomarker of resistance to CDK4/6i Potential predictive biomarker of resistance and poor clinical outcomes to CDK4/6i and/or ET Predictive biomarker of response to specific PI3KCAi	-	[29,65-68]
	Cyclin D (*CCND1*) (amplified)	Primary tumor: 20% Metastases: 17%	Potential prognostic biomarker (poor) Potential predictive biomarker of response to CDK4/6i	-	[32,69,70]
	Cyclin E (*CCNE1*) (amplified)	Primary tumor: NA Metastases: NA	Prognostic biomarker Predictive biomarker of resistance to CDK4/6i and or AI Predictive biomarker of response to CT (capecitabine)	-	[22,24,71-76]
	*TP53* (mutated)	Primary tumor: 18% Metastases: 28%	Prognostic biomarker (poor)	-	[4,69,70,77,78]

ABC: Advanced breast cancer; ADC: antibody-drug conjugate; AI: aromatase inhibitor; BRCA: breast cancer gene; CDK4/6i: cyclin-dependent kinase 4/6 inhibitors; CT: chemotherapy; ERS1: estrogen receptor 1; ET: endocrine therapy; HER2: human epidermal growth factor receptor 2; NA: not available; PARPi: PARP inhibitors; RB: retinoblastoma; SERD: estrogen receptor downregulator; SG: sacituzumab govitecan; T-DXd: trastuzumab deruxtecan; TKi: tyrosine kinase inhibitor.

## CLINICAL BIOMARKERS

In HR+/HER2- ABC, the level of ET sensitivity plays a critical role in determining the efficacy of first-line treatment with CDK4/6i combined with ET. The current criteria for classifying a patient as endocrine-sensitive or resistant are those established in the 5th ESMO-ABC Guidelines^[79]^. These consensus criteria classify patients into three groups:

**·** Primary ET resistance (1ET-R): defined as relapse within the first 2 years of adjuvant ET, or progressive disease (PD) within the first 6 months of first-line ET for ABC. **·** Secondary ET resistance (2ET-R): defined as relapse after the first 2 years of adjuvant ET, relapse occurring within 12 months of completing adjuvant ET, or PD ≥ 6 months after initiating ET for ABC. **·** ET sensitivity: patients relapsing after 12 months of completing adjuvant ET or presenting with *de novo* metastatic disease are considered to have ET-sensitive disease.

These criteria have demonstrated significant prognostic impact and are crucial for guiding first-line and subsequent treatment choices. Patients with ET-sensitive disease, characterized by relapse more than 12 months after completing adjuvant ET or presenting with *de novo* metastatic disease, achieve significantly better outcomes, including prolonged PFS and OS.

A comprehensive analysis of over 6,000 HR+/HER2- early BC patients across four phase III trials revealed critical insights. Among 493 patients with distant relapse, the mOS was 27.2 months for those with 1ET-R, 38.4 months for 2ET-R, and 43.2 months for ET-sensitive relapse^[80]^.

The AURORA study demonstrated significant differences in median PFS (mPFS) and OS (mOS) in first-line CDK4/6i + ET therapy based on adjuvant ET sensitivity ^[81]^. Similar findings were reported in the GEICAM_REGISTEM study, which assessed 800 HR+/HER2- primary tumors that relapsed following adjuvant ET. This study further confirmed significant differences in mPFS (months; *P* < 0.0001), which were also reflected in mOS (years; *P* = 0.02) across the same subgroups^[82]^. Key results from both studies, including mPFS and mOS values for patients with 1ET-R, 2ET-R, ET-sensitive relapses, and *de novo*/naïve ABC, are summarized in Table 2.

**Table 2:** mPFS and OS for HR+/HER2- ABC patients stratified by ET subgroups

**Subgroup**	**mPFS (months)**	**mOS (months/years)**
1ET-R	6.6-8.4	20.4/3.7
2ET-R	14.6-19.3	38.2/3.9
ET-sensitive relapse	26.3-27.9	55.6/4.85
*De novo*/naïve ABC	27.3	44.7

PFS: Progression-free survival; OS: overall survival; HR: hormone receptor; HER2: human epidermal growth factor receptor; ABC: advanced breast cancer; ET: endocrine therapy; 1ET-R: primary ET resistance; 2ET-R: secondary ET resistance; mOS: median overall survival; mPFS: median progression-free survival.

In alignment with these findings, the PARSIFAL LONG study reported consistent data underscoring the significance of ET-sensitive disease or *de novo* ABC as a prognostic biomarker associated with OS. Thus, patients who progressed after 12 months of first-line palbociclib therapy achieved a notable mOS of over 80 months, while those who progressed within 12 months had a shorter mOS (24 months)^[83]^. Furthermore, the GEICAM_REGISTEM study found that approximately 30% of patients with ET-sensitive relapse to adjuvant ET progressed during the first 12 months of first-line CDK4/6i + ET. The mOS was 1.2 years for those progressing within the first 6 months, 3.6 years for those progressing between 6-12 months, and 5.2 years for those progressing after 12 months (*P* < 0.001)^[82]^.

These findings highlight that defining ET sensitivity - whether based on the time to relapse following adjuvant ET or the response to first-line therapy for ABC - can stratify HR+/HER2- patients into distinct prognostic groups with varying sensitivity to CDK4/6 inhibitors. These definitions should be considered in clinical decision-making and clinical trial design to ensure homogeneous patient populations.

## ACTIONABLE GENOMIC BIOMARKERS

### PIK3CA

The *PIK3CA* gene encodes the catalytic subunit of phosphatidylinositol 3-kinase (PI3K). Mutations in this gene occur in 30%-40% of HR+/HER2- BC^[84]^, leading to hyperactivation of the canonical PI3K/AKT/mTOR pathway, which drives cell metabolism and proliferation. The prevalence of *PIK3CA* mutations may differ between primary tumors and metastases and can also be altered by treatment. While mutations were traditionally associated with better outcomes in early BC^[85]^, their prognostic value is poor in advanced stages^[34]^. Notably, *PIK3CA* mutations do not predict response to CDK4/6i^[5,86,87]^. In the PALOMA-3 study, circulating tumor DNA (ctDNA) sequencing identified the emergence of *PIK3CA* driver mutations during treatment. A relative change in *PIK3CA* ctDNA levels after 15 days of treatment strongly predicted PFS with palbociclib and fulvestrant^[28,29]^. Activation of the PI3K pathway is linked to resistance to ET in metastatic luminal BC. This finding led to the development of inhibitors targeting PI3K/AKT/mTOR pathway and their incorporation into clinical practice^[31,36,88]^.

Thus, the phase III SOLAR-1 trial comparing alpelisib plus fulvestrant to fulvestrant alone in HR+/HER2- ABC patients demonstrated a prolonged mPFS in those with *PIK3CA* mutations, without significant impact on OS^[31,33]^. Additionally, the phase II BYLieve trial highlighted the benefit of alpelisib plus fulvestrant after prior CDK4/6i (reporting a mPFS of 8 months)^[89]^. These findings, along with real-world data^[90,91]^, support the use of alpelisib in routine clinical practice after first-line CDK4/6i.

In the phase II FAKTION trial^[92]^ and the confirmatory phase III CAPITELLO-291 study^[93]^, capivasertib combined with fulvestrant significantly increased mPFS compared to placebo plus fulvestrant, following first-line CDK4/6i therapy in patients with alterations in the PI3K/AKT/PTEN pathway^[93-95]^.

Combinations of CDK4/6i with PI3K inhibitors (PI3Ki) and ET have been studied, though initial results indicated toxicity or limited efficacy^[96]^. To address these challenges, more selective PI3Ki are being investigated to minimize toxicity and enhance combination therapy approaches^[97,98]^. The phase III INAVO120 trial demonstrated improved PFS with the combination of inavolisib, palbociclib, and fulvestrant compared to fulvestrant plus palbociclib [15.0 months *vs.* 7.3 months, respectively; HR (hazard ratio): 0.43; 95%CI (confidence interval): 0.32-0.59] in patients with either primary or 2ET-R and mutated *PIK3CA*^[37,38]^. Based on these results, inavolisib was recently approved by the US Food and Drug Administration (FDA) for the first-line treatment of *PIK3CA*-mutated, HR+/HER2-, locally advanced or metastatic breast cancer. This approval marks a pivotal advancement in expanding therapeutic options for this subgroup of patients.

These findings highlight the importance of molecular testing to identify patients with HR+/HER2- ABC who may benefit from PI3K pathway inhibitors^[99]^. *PIK3CA* mutations, detectable through tumor tissue or ctDNA testing, are central to this approach. The concordance between tissue and blood genotyping is extremely high for tumors with a high DNA fraction, which generally makes ctDNA testing the preferred method. However, in cases of low ctDNA fraction or a negative result, tissue testing should be performed. Recent studies show that comprehensive genomic profiling detects a broader range of *PIK3CA* mutations beyond the standard SOLAR-1 trial set (which targets 11 mutations identified by the *therascreen*® kit), with approximately 20% of patients exhibiting non-SOLAR1 mutations. These patients may still benefit from alpelisib treatment, underscoring the importance of comprehensive genomic profiling for enhancing treatment personalization and improving patient outcomes^[100]^.

### ESR1

The *ESR1* gene encodes estrogen receptor (ER) alpha, a ligand-dependent transcription factor. Under therapeutic pressure, particularly from aromatase inhibitors (AI) in the metastatic setting, *ESR1* mutations markedly increase as a mechanism of resistance and are detected in up to 48% of pretreated patients^[101]^. The incidence of acquired *ESR1* mutations can reach up to 50% after one year of first-line treatment with CDK4/6i plus AI. However, when CDK4/6i in combination with fulvestrant is used as the first-line treatment, there appears to be no increase in *ESR1* mutations post-progression^[51]^. Activating mutations in the ER ligand-binding domain have significant clinical implications in HR+/HER2- ABC. These mutations are associated with AI resistance, greater sensitivity to fulvestrant in comparison to AI^[102]^, and greater benefit with new oral selective estrogen receptor downregulators (SERDs)^[43,103]^.

In the phase III SoFEA and EFECT trials, which explored the efficacy of ET monotherapy in the advanced setting, the detection of *ESR1* mutations in baseline ctDNA was associated with inferior PFS and OS in patients treated with exemestane *vs.* fulvestrant^[41]^. This suggested *ESR1* mutations as a potential biomarker for fulvestrant selection. A similar pattern was observed in patients receiving combined CDK4/6i with either AI or fulvestrant^[40,42]^. An exploratory analysis of the PADA-1 trial demonstrated that patients with *ESR1* mutations had a significantly shorter PFS than those without mutations, suggesting that baseline *ESR1* mutation could accelerate the onset of resistance to AI-palbociclib^[40]^. Furthermore, a recent study revealed that patients with *ESR1* mutation receiving first-line AI plus CDK4/6i had less favorable PFS and OS compared to those without *ESR1* mutation. However, no differences were observed in patients treated with fulvestrant plus CDK4/6i^[49,52]^. Thus, the presence of *ESR1* mutations confers resistance to AI plus CDK4/6i but not fulvestrant plus CDK4/6i. In fact, the PADA-1 trial demonstrated that switching from an AI to fulvestrant upon detecting an *ESR1* mutation, even in the absence of radiological progression, resulted in significant clinical benefits. This finding highlights the need for adaptive treatment strategies^[50]^. However, ctDNA sequencing at baseline and at the end of treatment in the PALOMA-3 trial revealed that some specific mutations, such as *ESR1* Y537S, also promoted acquired resistance to fulvestrant^[29]^. This detrimental effect appeared to be relatively overcome by new oral SERDs. In the EMERALD trial, elacestrant demonstrated a significant improvement in PFS compared to the standard-of-care, particularly in the *ESR1* mutated population^[43]^ and in the previously CDK4/6i sensitive population (more than 18 months)^[104]^. Similar results have been observed with other next-generation SERDs^[44,50]^, and new adaptive trials investigating this strategy are currently underway^[48]^.

A recommendation of *ESR1* mutation testing at recurrence or progression on ET in HR +/HER2- ABC has been incorporated into the latest American Society of Clinical Oncology (ASCO) guidelines^[105]^. Thus, the analysis of *ESR1* mutational status should be performed after progression to at least one ET line; plasma ctDNA measurement is the preferred testing method, given that *ESR1* mutations are frequently subclonal and polyclonal at progression and are better captured through ctDNA assays.

### *ERBB2* (*HER2*)

HER2-low expression in BC has been recently identified as a new therapeutic target. Approximately 45%-55% of HER2- ABC cases are HER2-low, defined by a score of 1+ or 2+ on immunohistochemical (IHC) analysis and negative *in situ* hybridization results^[106]^. The prevalence of HER2-low expression is approximately 34% in primary tumors and 37% in metastases, with an overall HER2 discordance rate of 40%-50% between primary tumors and matched distant metastases^[56,57]^. In this context, ET combined with a CDK4/6i remains an effective first-line treatment, irrespective of HER2 status (HER2-low/HER2-zero)^[107]^.

HER2-low BC is a biomarker for new antibody-drug conjugates (ADCs) such as trastuzumab deruxtecan (T-DXd). The DESTINY-Breast04 and DESTINY-Breast06 clinical trials have demonstrated significant benefits. The DESTINY-Breast04 trial showed superior PFS and OS with T-DXd compared to the physician’s choice of CT in HER2-low ABC patients, including those who had previously received CDK4/6i (70.4% of patients; mPFS of 10 months)^[55]^. Furthermore, the DESTINY-Breast06 trial has confirmed this benefit in patients who had received at least one endocrine-based therapy and had not received prior CT for metastatic breast cancer (MBC). The primary results showed significantly improved PFS with T-DXd compared to the physician’s choice of CT in HER2-low patients (13.2 months *vs.* 8.1 months, respectively; HR: 0.62; 95%CI: 0.51, 0.74)^[58]^, including patients with 1ET-R (13.1 months *vs*. 6.8 months; HR: 0.56, 95%CI: 0.40, 0.78)^[59]^. More recent studies have further explored the implications of HER2-low status in ABC. The DEBBRAH trial highlighted the intracranial activity of T-DXd in HER2-low ABC patients with active brain metastases, demonstrating promising response rates in heavily pretreated individuals^[108]^. In addition, recent findings suggest that resistance to T-DXd in HER2-low BC may involve circular RNA (crVDAC3). Targeting this pathway has shown potential in restoring T-DXd sensitivity, offering a new strategy to enhance treatment outcomes in HER2-low ABC patients^[109]^.

*ERBB2* mutations are rare but more prevalent in invasive lobular carcinoma. Mutations comprise hot-spot activating missense mutations (e.g. S310F/Y, L755S and V777L) and in-frame insertions exon 20 (Ex: Y772_A775dup) which occurs in 2%-4% of BC patients^[110,111]^. In luminal ABC, *HER2* activating mutations are likely acquired under the selective pressure of ER-targeted treatments (including ET alone or combined with CDK4/6i), as previously described^[112]^. In the phase II SUMMIT basket trial, neratinib-based therapy, specifically the combination of neratinib + fulvestrant + trastuzumab (N + F + T), provided HR+ HER2/3-mutant MBC patients with an objective response rate (ORR) of 39% and a mPFS of 8.3 months, all of whom had progressed after prior CDK4/6i. The ORR in ductal and lobular MBC with ≥ 1 HER2 mutation or concomitant HER3 mutation was 39% and 41%, respectively^[53]^.

### *BRCA1* and *BRCA2*

Somatic (s) and germline (g) alterations of tumor suppressors *BRCA1* and *BRCA2* are linked to homologous recombination deficiency (HRD) with implications for cancer inheritance^[113]^. Pathogenic variants (PV) of *gBRCA1*, *BRCA2*, and other HRD-associated genes have prognostic significance in BC and correlate independently with poor outcomes in CDK4/6i-treated patients^[114,115]^. Real-world evidence^[116]^ suggested that HR+ ABC patients with *BRCA1* and *BRCA2* PV had a worse prognosis with palbociclib , as demonstrated in the exploratory analyses of the randomized phase II Young Pearl study for *BRCA2*^[117]^.

Poly ADP-ribose polymerase inhibitors (PARPi) such as olaparib or talazoparib demonstrated superiority to CT in HER2- ABC with *gBRCA1/2* mutations in terms of PFS but not OS^[60-63,118]^. The phase III OlympiAD trial showed that olaparib significantly improved PFS compared to CT (7.0 months *vs.* 4.2 months; HR: 0.58; *P* < 0.001) in patients with HER2- ABC and *gBRCA1/2* mutations^[61]^. Similarly, the phase III trial EMBRACA demonstrated a benefit of talazoparib in this population with a mPFS of 8.6 months compared to 5.6 months with CT (HR: 0.54; *P* < 0.001)^[60]^. These findings underscore the importance of identifying *BRCA1/2* mutations to guide treatment strategies. Although both trials included HR+ and triple-negative BC patients, subgroup analyses suggested similar benefits for PARPi in HR+/HER2- ABC. Additionally, the TBCRC 048 phase II study reported promising ORR and PFS with olaparib in HER2- ABC with *gPALB2* and *sBRCA1/2* mutations^[64]^.

There is a lack of evidence regarding PARPi efficacy after CDK4/6i, but the poor prognostic impact of *BRCA1* and *BRCA2* alterations (and other mutated HRD-genes) in CDK4/6i responses has prompted the search for alternatives to circumvent resistance. Current Clinical Practice Guidelines (CPG) recommend PARPi treatment for *gBRCA1/2* and as an option for *sBRCA1/2* and *gPALB2* mutations^[15,119]^ and, consequently, testing for HRD-gene alterations to guide treatment decisions^[79]^. Analysis of *gBRCA1* or *BRCA2* status can be conducted through next-generation sequencing (NGS) on blood, saliva, or tumor tissue^[120]^.

## NON-ACTIONABLE BIOMARKERS

### Retinoblastoma

Retinoblastoma (RB) is a tumor suppressor protein encoded by the *RB1* gene and plays a crucial role in cell cycle regulation^[121]^. In tumors reliant on the cyclin D1-CDK4/6-RB axis for growth, treatment with *CDK4/6i* reduces RB phosphorylation and induces cell cycle arrest. The loss of RB function represents a specific vulnerability for therapeutic intervention as it has been clearly associated with ET and CDK4/6i resistance in *in vitro* analyses^[122]^. Emerging evidence suggests that this phenomenon could extend to the clinical context as well^[29,65,67,68]^, although current CPG do not restrict the use of CDK4/6i solely based on pathogenic *RB1* mutations. *RB1* alterations are rare in CDK4/6i-naïve BC patients (0%-3%) but have been detected in up to 11.5% upon progression to CDK4/6i treatment, and are commonly associated with subclonal variants^[65,123]^.

Several genetic anomalies linked to RB loss of function include complete or partial loss of alleles, gene deletions, promoter methylation, and minor inter- or intragenic mutations, whose detection is challenging and hampered by technical limitations in the clinical setting. The assessment of copy number losses, particularly through liquid biopsy assays, lacks sufficient sensitivity to be routinely adopted^[124]^. Furthermore, identifying these alterations through metastatic tissue analysis can be complicated due to their polyclonality and intertumor heterogeneity. On the other hand, the detection of RB loss through IHC or mRNA expression analyses in BC samples from clinical trials has failed to predict response to CDK4/6i^[23,125]^. Notwithstanding, gene-expression signatures focusing on inactivation of the RB pathway have been shown to be prognostic in BC as well as potentially predictive of response to CDK4/6i^[126]^.

### Cyclin D

The cell cycle regulator cyclin D1, encoded by the *CCND1* oncogene, serves as a common downstream effector of different proliferation signals, converging at the nuclear level through the allosteric activation of CDK4/6 and, subsequently, RB. Cancers with cyclin D activation have shown particular sensitivity to CDK4/6i^[127]^. Clinical trials revealed that tumors with *CCND1* amplification did derive significant benefits from palbociclib relative to those without amplification^[128]^. *CDK6* gene amplification results in marked *CDK6* overexpression in abemaciclib-resistant HR+ and palbociclib-treated ER+ BC, as shown in *in vitro* analyses^[129,130]^. However, increased *CDK6* mRNA expression has not been associated with resistance to CDK4/6i in clinical samples^[23,24]^.

### CDK2-Cyclin E signaling

*CCNE1* encodes cyclin E, which, upon binding to CDK2, regulates S phase entry^[131]^. Cyclin E1 amplification and overexpression are associated with poor prognosis in BC^[132]^, while *CCNE1* amplifications^[71,133]^, cyclin E1 upregulation^[73]^, and an elevated *CCNE1/RB1* ratio^[74]^ correlate with CDK4/6i resistance. Various preclinical studies^[74,96,134]^ and subgroup analyses from the PALOMA-3 and PEARL clinical trials^[22,24]^ found that high *CCNE1* mRNA levels predicted palbociclib resistance, although nuclear cyclin E1 analysis does not specifically predict CDK4/6i resistance^[135]^. Cytoplasmic cyclin E protein, indicative of low-molecular-weight cyclin E isoforms, is a biomarker of aggressive BC, potential resistance to AIs and CDK4/6i^[75,76]^, and increased sensitivity to capecitabine^[136]^.

Studies of cell lines and gene dependencies have shown that BC cells that are *RB1*-proficient but not dependent on CDK4/6, rely heavily on CDK2 and cyclin E1 for survival and proliferation. Therefore, CDK2 inhibition represents a promising therapeutic alternative for cancers with CDK2/cyclin E-dependency^[137,138]^.

### TP53

*TP53* encodes the P53 protein, a critical tumor suppressor that responds to cellular stress by regulating various cellular processes, leading to cell cycle arrest, apoptosis, DNA repair, and metabolic changes. *TP53* mutations are strongly associated with primary endocrine resistance in early HR+ BC^[139]^ and advanced disease, where they are linked to poor prognosis, irrespective of ET or CT^[77]^. However, there is currently no validated predictive value for treatment selection based on *TP53* mutations. In the MONALEESA-7 trial, ribociclib demonstrated similar efficacy irrespective of *TP53* status, although *TP53*-altered tumors had shorter PFS^[32]^. Similarly, the PALOMA-3 trial revealed that palbociclib plus fulvestrant was associated with more favorable PFS and OS outcomes compared to placebo plus fulvestrant, regardless of mutations in *ESR1*, *PIK3CA*, or *TP53*, although a better prognosis was observed in patients without mutations. High circulating tumor fraction was associated with worse PFS^[4,78]^. While *TP53* mutations are not actionable targets in standard practice, they should be included in NGS panels for prognostic purposes, especially in clinical research centers conducting ongoing clinical trials with available targeted treatments.

## RESISTANCE: WHICH ARE THE BEST BIOMARKERS AND WHICH ARE THE OPTIONS

**Figure 1:**
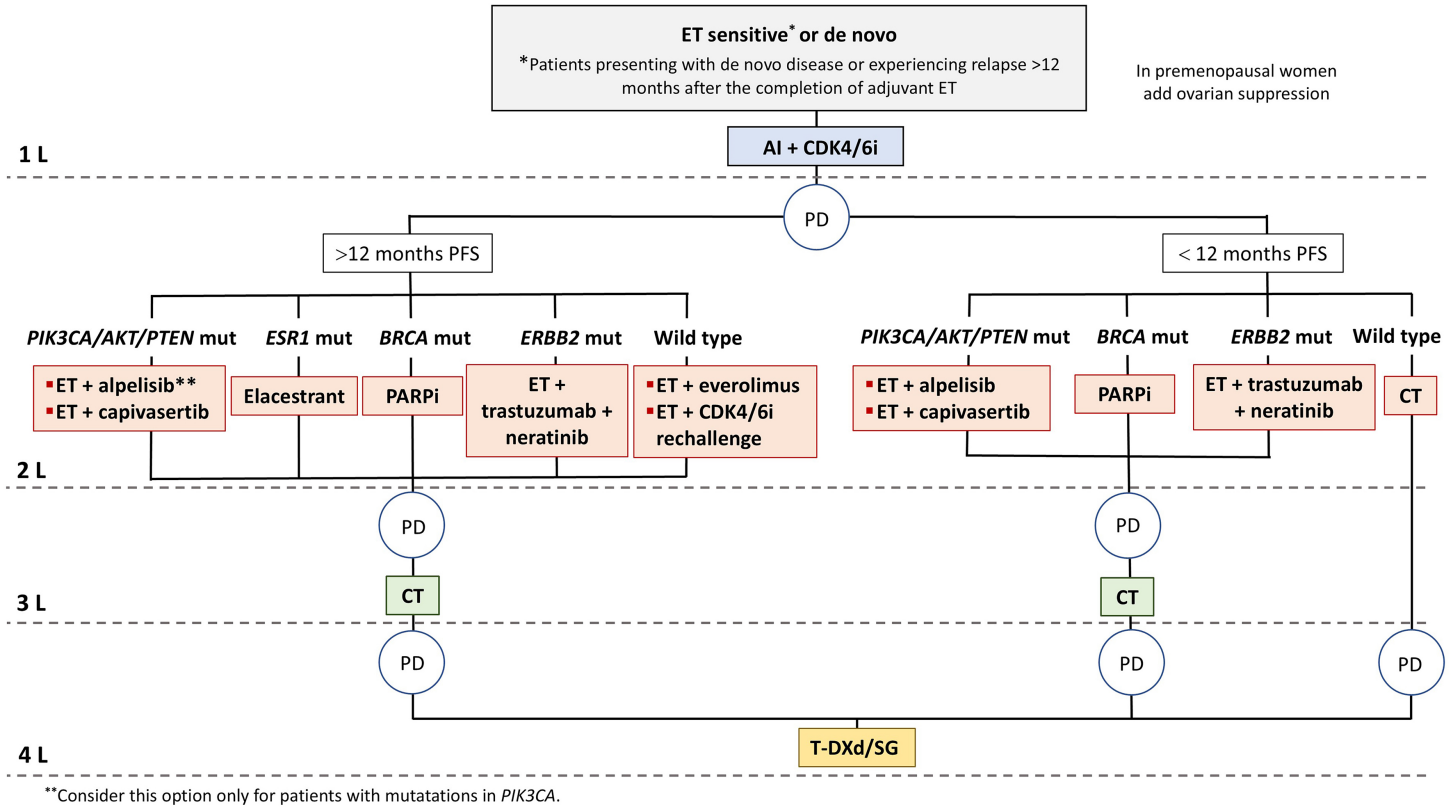
Management of endocrine-sensitive or *de novo* luminal ABC patients. For luminal ABC, endocrine-sensitive with or without targeted therapy remains the mainstay of treatment. Prior lines of therapy should be exhausted before initiating CT. Preferred therapeutic options are represented for women harboring specific gene mutations or without identified mutations. AI: Aromatase inhibitor; BRCA: breast cancer gene; CDK4/6i: cyclin-dependent kinase 4/6 inhibitors; CT: chemotherapy; ERS1: estrogen receptor 1; ET: endocrine therapy; ERBB2: v-erb-b2 avian erythroblastic leukemia viral oncogene homolog 2; mut: mutated; PD: progressive disease; PFS: progression-free survival; SG: Sacituzumab govitecan; T-DXd: trastuzumab deruxtecan; wt: wild type; ABC: advanced breast cancer.

**Figure 2:**
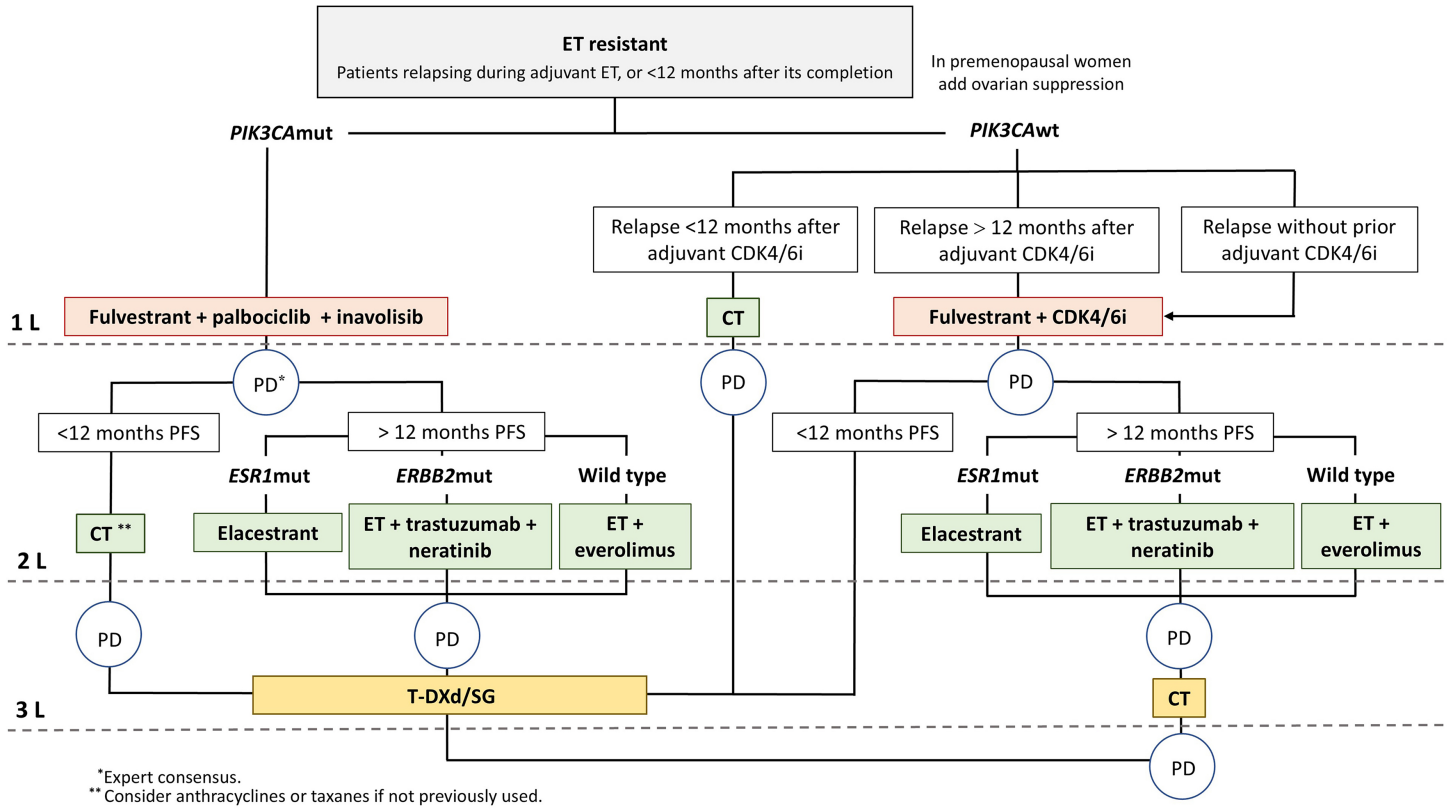
Management of endocrine-resistant luminal ABC patients. Similar to the endocrine-sensitive scenario, endocrine-resistant with or without targeted therapy remains a core treatment approach. Prior lines of therapy should be used before initiating CT. Preferred therapeutic options are represented for women harboring specific gene mutations or without identified mutations. All therapies included are evidence-based unless otherwise noted. AI: Aromatase inhibitor; BRCA: breast cancer gene; CDK4/6i: cyclin-dependent kinase 4/6 inhibitors; CT: chemotherapy; ERS1: estrogen receptor 1; ET: endocrine therapy; ERBB2: v-erb-b2 avian erythroblastic leukemia viral oncogene homolog 2; mut: mutated; PD: progressive disease; PFS: progression-free survival; SG: Sacituzumab govitecan; T-DXd: trastuzumab deruxtecan; wt: wild type; ABC: advanced breast cancer.

Combined ET and CDK4/6i should be a first-line treatment for most HR+/HER2- ABC patients [therapeutic algorithms are shown in Figures 1 and 2], given the clinically meaningful benefits in PFS and OS, manageable toxicity, and maintenance or improvement in QoL observed in several phase III trials^[2,6,140-142]^, and in line with current CPG recommendations^[15,143]^. Re-biopsy of metastases at recurrence, if feasible, is recommended in CPG^[15,143]^ due to intrinsic subtype changes and inconsistent HER2 expression. NGS performed on tumor tissue or liquid biopsy in metastatic disease is essential for comprehensive molecular profiling, including *ESR1* and *PIK3CA* gene assessments, among others. NGS is typically utilized in the second-line setting following progression on ET and CDK4/6i. However, findings from the recent INAVO-120 trial (NCT04191499) and the identification of early-relapsing patients post-adjuvant CDK4/6i therapy, coupled with the increased frequency of *ESR1* mutations reported in the AURORA trial^[81]^, might support considering earlier NGS implementation, at least in the first-line setting for endocrine-resistant disease. Therapeutic decisions are complex upon disease progression following first-line ET + CDK4/6i. There is still a limited understanding of predominant resistance mechanisms and the availability of clinically validated biomarkers.

Below we outline potential therapeutic options and highlight useful biomarkers to consider in the post-CDK4/6i setting. In addition, a summary of umbrella clinical trials exploring second-line treatments in this setting is provided in Supplementary Table 1.

(1) Switching ET and discontinuing CDK4/6i. The use of novel oral SERD in this setting is supported by the results of the EMERALD trial, which demonstrated improvements in PFS and QoL with elacestrant *vs.* standard of care (SOC). In patients with mutated *ESR1*, the duration of prior CDK4/6i was a potential predictor of efficacy, as a longer duration of prior CDK4/6i therapy was positively associated with prolonged PFS with elacestrant *vs.* SOC^[43]^. The trials SERENA-2 with camizestrant^[44]^, acelERA with giredestrant^[45]^, and AMEERA-3 with amcenestrant^[35]^ have also supported this approach.

Monotherapy with a selective proteolysis-targeting chimera protein degrader (ARV-471) in the phase II dose-expansion VERITAC study showed an enhanced clinical benefit rate (CBR) in HR+/HER2- advanced/MBC, particularly in patients with *ESR1* mutation^[46]^. The phase III VERITAC 2 study (ClinicalTrials.gov NCT05654623) is evaluating ARV-471 *vs.* fulvestrant at progression after CDK4/6i.

(2) Switching ET upon progression and continuing CDK4/6 inhibition has been particularly explored with palbociclib. The PADA-1 trial also suggested the benefit of early switching to a SERD at molecular progression upon detection of a rising *ESR1* mutation in ctDNA^[50]^ while awaiting further validation. The phase III SERENA-6 (NCT04964934) trial is evaluating the efficacy and safety of switching from AI to camizestrant while maintaining the same CDK4/6i^[48]^. The phase II PACE^[123]^ and phase III PALMIRA studies^[144]^ showed no PFS benefit with palbociclib continuation after progression. Conversely, the phase II MAINTAIN trial suggested switching CDK4/6i, as patients previously treated with palbociclib experienced improved PFS with ribociclib^[145]^. This could be due to the different CDK4/6i dosing received after progression in each of the trials. While patients in the PALMIRA and PACE trials were re-treated at progression with the same palbociclib dose, patients in the MAINTAIN trial started ribociclib at the highest dose (600 mg) at progression on palbociclib. Observational data support switching to abemaciclib^[133,146,147]^, even upon detection of mutated *ERS1*^[148]^. This is further validated by the post-MONARCH phase 3 trial, which showed a significant PFS improvement with abemaciclib plus fulvestrant compared to placebo plus fulvestrant in HR+/HER2- ABC at progression after CDK4/6i + ET (HR: 0.73; 95%CI: 0.57, 0.95 ), with PFS rates at 6 months of 50% *vs.* 37%, respectively. The effect was consistent across major subgroups, including patients with baseline *ESR1* or *PIK3CA* mutations^[149]^.

No validated biomarkers are available to guide decisions in this scenario. However, in the MAINTAIN trial, *ESR1* or *PIK3CA* mutations were associated with a lack of benefit after switching from first-line-palbociclib to ribociclib after progression^[145]^, consistent with the clonal evolution observed in patients with late progression in the PALOMA-3 trial^[29,150]^. The BIOPER study identified RB loss and increased cyclin E as markers of resistance^[125]^.

(3) Targeting the PI3K/AKT/mTOR pathway post-progression to first-line CDK4/6i. Three groups of drugs are currently available:

· mTOR inhibitors: Everolimus plus exemestane showed improved PFS in CDK4/6i naïve patients ^[151]^, with similar benefits after CDK4/6i according to observational data^[152]^. · PI3K inhibitors: Alpelisib plus fulvestrant *vs.* fulvestrant demonstrated prolonged PFS in patients with *PIK3CA*, as supported by the SOLAR-1 and BYLieve trials^[31]^. Data from the BYLieve trial and real-world evidence^[89-91]^ confirmed the utility of PI3Ki as a next-line treatment following CDK4/6i or even everolimus^[153]^. · AKT inhibitors: The AKT inhibitor capivasertib, approved for use with fulvestrant as a second-line therapy option following first-line CDK4/6i, demonstrated increased PFS in patients with *AKT*, *PTEN*, and *PIK3CA* alterations, as confirmed by the phase II FAKTION trial^[92,94]^ and the confirmatory phase III CAPITELLO-291 study^[93]^.

(4) ADC. T-DXd and sacituzumab govitecan (SG) have shown significant survival benefits in HR+/HER2- and HER2-low BC patients, respectively, refractory to ET^[55,154]^. Although restricted to patients with at least one prior CT line, ADC therapy is impacting luminal BC outcomes^[35,50,55,155]^, offering innovative treatment options. Several ADCs are currently under development, such as datopotamab deruxtecan and patritumab deruxtecan^[44]^. HER2 is the sole validated biomarker for identifying HER2-low tumors for T-DXd treatment^[55]^. Trop-2 expression is not deemed a reliable predictive marker for survival benefit with SG^[156]^.

Considering all the above, in the context of resistance to first-line combined treatment with ET plus CDK4/6i, biomarker determination is highly valuable, with a high level of evidence (ESCAT I)^[157]^. Current CPG recommend the profiling of *gBRCA1/2* and *PIK3CA* mutations^[99,143]^. Additionally, the identification of other ESCAT II alterations, such as *ESR1* and *AKT1* mutations, may also be considered.

The clinical scenarios should guide both the indication and timing for determining these biomarkers and the criteria for making decisions based on their results.

## RECOMMENDATIONS: FROM EVIDENCE TO CLINICAL PRACTICE

The following specific recommendations can be considered in the setting of progression to first-line treatment:

· Testing for *gBRCA1/2* mutations is recommended for ABC patients who are potentially eligible for PARP inhibition after ET failure. · *PIK3CA* mutations should be tested in patients progressing ≥ 6 months on first-line treatment for consideration of second-line alpelisib. Screening for *AKT*, *PIK3CA*, and *PTEN* mutations is advisable to evaluate eligibility for capivasertib. · *ESR1* mutations should be determined by liquid biopsy in patients on first-line AI-based ET progressing at ≥ 6 months (preferably ≥ 12 months) to guide treatment with elacestrant or other SERD. · Although no clear biomarkers are available to identify candidates for switching CDK4/6i, the absence of both *PIK3CA* and *ESR1* mutations might support this strategy, especially with slow disease progression at first-line (≥ 12 months). Patients without *PIK3CA*/*ESR1* mutations may also benefit from ET plus everolimus. · Finally, NGS with extensive gene panels is recommended only in clinical research centers evaluating targeted treatments within clinical trials. The likelihood of finding genomic alterations indicating specific or agnostic molecular targets in luminal ABC is low, and the clinical utility of ET resistance markers such as *TP53* mutations is yet to be demonstrated^[136,139]^. Furthermore, performance in the identification of PV in g DNA was poorer^[158]^. However, the recent incorporation of *ESR1* and *PIK3CA* mutations as validated markers fulfills other guidelines, suggesting that in instances where multiple biomarker-targeted therapies are approved for the patient’s condition, multigene panel-based assays should be employed^[159]^. Thus, current guidelines in this setting may evolve in the future.

## UPCOMING BIOMARKERS AND FUTURE THERAPEUTIC DIRECTIONS

Despite the significant advancements achieved with CDK4/6i in HR+/HER2- ABC, several limitations persist. These include primary and acquired resistance, which ultimately limit their long-term efficacy in a substantial proportion of patients. Mechanisms of resistance, such as cyclin E overexpression, *RB1* mutations, and bypass signaling pathways, highlight the complexity of tumor biology and the need for alternative therapeutic strategies. The toxicity associated with combination therapies, often required to address multiple mechanisms of resistance, presents an additional barrier, limiting their tolerability and feasibility. These challenges underscore the urgent need to identify novel biomarkers and develop next-generation targeted therapies to overcome resistance and improve outcomes.

### CDK7

Cyclin-dependent kinase 7 (CDK7) regulates transcription and the cell cycle and, once phosphorylated, activates estrogen and androgen receptors^[160]^. CDK7 overexpression in BC leads to CDK4/6i resistance and poor prognosis^[161,162]^. Preclinical studies have demonstrated the sensitivity of many cancers to novel selective CDK7i^[163]^. Recently, samuraciclib, an oral CDK-7i, has demonstrated clinical activity in a phase I clinical trial, particularly in luminal BC patients with no *TP53* mutations, with a CBR of 47.4%^[164]^.

### HRD and *PALB2* mutations

Loss-of-function mutations in genes involved in homologous recombination repair (HRR) can sensitize tumors to double-strand break (DSB)-triggering agents such as PARPi and platinum-based chemotherapy (PT-CT), exploiting synthetic lethality through HRD ^[165]^. Besides *BRCA1* and *BRCA2*, *PALB2* or *RAD51D* alterations in s or g cells are associated with HRD. In addition, HRD assessment through the genomic HRD score (e.g. Myriad myChoice) or functional tests like RAD51 foci has proven to be more effective in predicting response to PARPi or PT-CT in HER2- BC^[166,167]^.

### The next generation of CDKi: beyond CDK4/6i

*CDK4* expression in BC samples is higher than *CDK6,* suggesting a pivotal role for CDK4 in BC cell proliferation, as these cells depend more on CDK4 than CDK6 for growth^[74,168]^. The CDK4 to CDK6 inhibition ratio could influence drug sensitivity and resistance. Preclinical studies indicate that palbociclib binds similarly to CDK4/cyclin D3 and CDK6/cyclin D complexes, while ribociclib and abemaciclib show a higher affinity for CDK4/cyclin D3^[74]^. The relatively lower inhibition of CDK6 by abemaciclib, compared to palbociclib or ribociclib, may explain its lower incidence of myelosuppression, enabling continuous administration.

PF-07220060, a potent, selective CDK4 inhibitor in early development, is likely less myelotoxic than currently approved CDK4/6i, and has shown promising activity in combination with ET after progression on previous CDK4/6i^[169]^.

Future therapeutic strategies are focusing on the development of specific CDK2 inhibitors (CDK2i). Cyclin E is a predictor of poorer OS and response to CDK2i and is associated with CDK4/6i resistance. PF-07104091, a novel selective CDK2i, has demonstrated good tolerance and antitumor activity in heavily pretreated HR+/HER2- ABC patients who have progressed on previous CDK4/6i^[170]^. Preclinical studies suggest that co-inhibition of CDK2 and CDK4/6 may be necessary to overcome intrinsic cell-cycle plasticity^[171,172]^. An ongoing phase I/IIB trial is currently evaluating the combination of CDK2 and CDK4 inhibition in advanced solid tumors, including BC (NCT05262400).

### KAT6

KAT6 is a histone lysine acetyltransferase that regulates lineage-specific gene transcription through H3K23 acetylation. A recent phase I study evaluating PF-07248144, a novel selective catalytic inhibitor of KAT6A and KAT6B, showed a tolerable safety profile and durable efficacy in heavily pretreated HR+/HER2- MBC patients, both with and without *ESR1* or *PIK3CA/AKT1/PTEN* mutations^[173]^.

In summary, the upcoming biomarkers and next-generation therapeutic strategies discussed above offer promising solutions to the limitations of current CDK4/6i. CDK7i, such as samuraciclib, target transcriptional dependencies and bypass mechanisms, thereby addressing resistance linked to transcriptional plasticity. Biomarkers like HRD scores and *PALB2* mutations enhance patient stratification for PARPi or platinum-based therapies, optimizing treatment selection. Meanwhile, selective CDK4i and CDK2i aim to refine the specificity of cell-cycle targeting, reducing toxicity and overcoming resistance mechanisms such as cyclin E overexpression. Collectively, these advancements not only expand therapeutic options but also hold the potential to significantly improve patient outcomes by addressing both efficacy and tolerability challenges.

Advances in artificial intelligence, particularly deep learning (DL), have shown significant potential in BC diagnosis, prognosis, and treatment response prediction^[174]^. DL has also emerged as a transformative tool for identifying cancer biomarkers by integrating multi-omics data and imaging features, enabling patient stratification and therapy response prediction ^[175,176]^. For instance, a machine learning model using clinic-pathological features was developed to identify HR+/HER2- ABC patients likely to respond poorly to first-line CDK4/6i, showcasing its utility for guiding personalized clinical interventions^[177]^. Furthermore, such approaches hold promise for guiding second-line treatment decisions in HR+/HER2- ABC after the failure of ET and CDK4/6 inhibitors by identifying biomarkers predictive of therapeutic response.

## CONCLUSIONS

In summary, the approval of CDK4/6i represented a significant advancement in the treatment paradigm for ER+/HER2- ABC, supported by robust evidence from phase III trials demonstrating improved PFS and OS outcomes when combined with ET. Key trials have reported mPFS values ranging from 14.8 to 26.7 months and median OS extending up to 53.7 months. Despite their efficacy, challenges such as variable drug response and the development of resistance mechanisms persist, highlighting the need for further research into personalized treatment strategies. Integrating identified biomarkers into clinical practice is crucial for tailoring therapeutic approaches and optimizing patient outcomes. For instance, therapies like alpelisib have demonstrated a mPFS improvement of up to 11 months in patients with *PIK3CA* mutations. Moreover, the ongoing exploration of alternative therapeutic options for refractory patients underscores the continuous efforts to enhance treatment efficacy. With emerging biomarkers alongside advancements in next-generation CDKi and targeted therapies, there is tangible optimism for the future of ER+/HER2- BC management. Moreover, the use of NGS panel tests utilizing tissue and/or blood has the potential to provide clues for overcoming drug resistance and improve tailored treatment.

## DECLARATIONS

### Copyright

© The Author(s) 2025.

## References

[1] Spring LM, Wander SA, Andre F, Moy B, Turner NC, Bardia A (2020). Cyclin-dependent kinase 4 and 6 inhibitors for hormone receptor-positive breast cancer: past, present, and future. Lancet.

[2] Finn RS, Crown JP, Ettl J (2016). Efficacy and safety of palbociclib in combination with letrozole as first-line treatment of ER-positive, HER2-negative, advanced breast cancer: expanded analyses of subgroups from the randomized pivotal trial PALOMA-1/TRIO-18. Breast Cancer Res.

[3] Rugo HS, Finn RS, Diéras V (2019). Palbociclib plus letrozole as first-line therapy in estrogen receptor-positive/human epidermal growth factor receptor 2-negative advanced breast cancer with extended follow-up. Breast Cancer Res Treat.

[5] Turner NC, Slamon DJ, Ro J (2018). Overall survival with palbociclib and fulvestrant in advanced breast cancer. N Engl J Med.

[6] Goetz MP, Toi M, Campone M (2017). MONARCH 3: abemaciclib as initial therapy for advanced breast cancer. J Clin Oncol.

[7] Johnston S, Martin M, Di Leo A (2019). MONARCH 3 final PFS: a randomized study of abemaciclib as initial therapy for advanced breast cancer. NPJ Breast Cancer.

[8] Sledge GW Jr, Toi M, Neven P (2020). The effect of abemaciclib plus fulvestrant on overall survival in hormone receptor-positive, ERBB2-negative breast cancer that progressed on endocrine therapy-MONARCH 2: a randomized clinical trial. JAMA Oncol.

[9] Sledge GW Jr, Toi M, Neven P (2017). MONARCH 2: abemaciclib in combination with fulvestrant in women with HR+/HER2- advanced breast cancer who had progressed while receiving endocrine therapy. J Clin Oncol.

[10] Cristofanilli M, Rugo HS, Im S (2021). Overall survival (OS) with palbociclib (PAL) + fulvestrant (FUL) in women with hormone receptor-positive (HR+), human epidermal growth factor receptor 2-negative (HER2-) advanced breast cancer (ABC): updated analyses from PALOMA-3. JCO.

[11] Im SA, Lu YS, Bardia A (2019). Overall survival with ribociclib plus endocrine therapy in breast cancer. N Engl J Med.

[12] Slamon DJ, Neven P, Chia S (2020). Overall survival with ribociclib plus fulvestrant in advanced breast cancer. N Engl J Med.

[13] Slamon DJ, Neven P, Chia SKL (2021). Updated overall survival (OS) results from the phase III MONALEESA-3 trial of postmenopausal patients (pts) with HR+/HER2- advanced breast cancer (ABC) treated with fulvestrant (FUL) ± ribociclib (RIB). JCO.

[14] Lloyd MR, Spring LM, Bardia A, Wander SA (2022). Mechanisms of resistance to CDK4/6 blockade in advanced hormone receptor-positive, HER2-negative breast cancer and emerging therapeutic opportunities. Clin Cancer Res.

[16] Perou CM, Sørlie T, Eisen MB (2000). Molecular portraits of human breast tumours. Nature.

[17] Holm J, Yu NY, Johansson A (2021). Concordance of Immunohistochemistry-based and gene expression-based subtyping in breast cancer. JNCI Cancer Spectr.

[18] Cejalvo JM, Pascual T, Fernández-Martínez A (2018). Clinical implications of the non-luminal intrinsic subtypes in hormone receptor-positive breast cancer. Cancer Treat Rev.

[19] Prat A, Cheang MC, Galván P (2016). Prognostic value of intrinsic subtypes in hormone receptor-positive metastatic breast cancer treated with letrozole with or without lapatinib. JAMA Oncol.

[20] Cejalvo JM, Martínez de Dueñas E, Galván P (2017). Intrinsic subtypes and gene expression profiles in primary and metastatic breast cancer. Cancer Res.

[21] Prat A, Brase JC, Cheng Y (2019). Everolimus plus exemestane for hormone receptor-positive advanced breast cancer: a PAM50 intrinsic subtype analysis of BOLERO-2. Oncologist.

[22] Turner NC, Liu Y, Zhu Z (2019). Cyclin E1 expression and palbociclib efficacy in previously treated hormone receptor-positive metastatic breast cancer. J Clin Oncol.

[23] Finn RS, Liu Y, Zhu Z (2020). Biomarker analyses of response to cyclin-dependent kinase 4/6 inhibition and endocrine therapy in women with treatment-naïve metastatic breast cancer. Clin Cancer Res.

[24] Guerrero-Zotano Á, Belli S, Zielinski C (2023). CCNE1 and PLK1 mediate resistance to palbociclib in HR+/HER2- metastatic breast cancer. Clin Cancer Res.

[25] Prat A, Chaudhury A, Solovieff N (2021). Correlative biomarker analysis of intrinsic subtypes and efficacy across the MONALEESA phase III studies. J Clin Oncol.

[26] Aftimos P, Oliveira M, Irrthum A (2021). Genomic and transcriptomic analyses of breast cancer primaries and matched metastases in AURORA, the breast international group (BIG) molecular screening initiative. Cancer Discov.

[27] Dupont Jensen J, Laenkholm AV, Knoop A (2011). PIK3CA mutations may be discordant between primary and corresponding metastatic disease in breast cancer. Clin Cancer Res.

[28] O'Leary B, Hrebien S, Morden JP (2018). Early circulating tumor DNA dynamics and clonal selection with palbociclib and fulvestrant for breast cancer. Nat Commun.

[29] O'Leary B, Cutts RJ, Liu Y (2018). The genetic landscape and clonal evolution of breast cancer resistance to palbociclib plus fulvestrant in the PALOMA-3 trial. Cancer Discov.

[30] Juric D, Ciruelos E, Rubovszky G (2019). Abstract GS3-08: alpelisib + fulvestrant for advanced breast cancer: subgroup analyses from the phase III SOLAR-1 trial. Cancer Research.

[32] Bardia A, Su F, Solovieff N (2021). Genomic profiling of premenopausal HR+ and HER2- metastatic breast cancer by circulating tumor DNA and association of genetic alterations with therapeutic response to endocrine therapy and ribociclib. JCO Precis Oncol.

[33] André F, Ciruelos EM, Juric D (2021). Alpelisib plus fulvestrant for PIK3CA-mutated, hormone receptor-positive, human epidermal growth factor receptor-2-negative advanced breast cancer: final overall survival results from SOLAR-1. Ann Oncol.

[34] Fillbrunn M, Signorovitch J, André F (2022). PIK3CA mutation status, progression and survival in advanced HR + /HER2- breast cancer: a meta-analysis of published clinical trials. BMC Cancer.

[35] Tolaney SM, Bardia A, Marmé F (2023). Final overall survival (OS) analysis from the phase 3 TROPiCS-02 study of sacituzumab govitecan (SG) in patients (pts) with hormone receptor–positive/HER2-negative (HR+/HER2-) metastatic breast cancer (mBC). JCO.

[36] Dent S, Cortés J, Im YH (2021). Phase III randomized study of taselisib or placebo with fulvestrant in estrogen receptor-positive, PIK3CA-mutant, HER2-negative, advanced breast cancer: the SANDPIPER trial. Ann Oncol.

[37] Hong R, Edgar K, Song K (2018). Abstract PD4-14: GDC-0077 is a selective PI3Kalpha inhibitor that demonstrates robust efficacy in *PIK3CA* mutant breast cancer models as a single agent and in combination with standard of care therapies. Cancer Research.

[38] Turner NC, Im SA, Saura C (2024). Inavolisib-based therapy in *PIK3CA*-mutated advanced breast cancer. N Engl J Med.

[39] Jeselsohn R, Yelensky R, Buchwalter G (2014). Emergence of constitutively active estrogen receptor-α mutations in pretreated advanced estrogen receptor-positive breast cancer. Clin Cancer Res.

[40] Bidard FC, Callens C, Dalenc F (2020). Prognostic impact of ESR1 mutations in ER+ HER2- MBC patients prior treated with first line AI and palbociclib: an exploratory analysis of the PADA-1 trial. JCO.

[41] Turner NC, Swift C, Kilburn L (2020). *ESR1* Mutations and overall survival on fulvestrant versus exemestane in advanced hormone receptor-positive breast cancer: a combined analysis of the phase III SoFEA and EFECT trials. Clin Cancer Res.

[42] Martin M, Zielinski C, Ruiz-Borrego M (2021). Palbociclib in combination with endocrine therapy versus capecitabine in hormonal receptor-positive, human epidermal growth factor 2-negative, aromatase inhibitor-resistant metastatic breast cancer: a phase III randomised controlled trial-PEARL. Ann Oncol.

[43] Bidard FC, Kaklamani VG, Neven P (2022). Elacestrant (oral selective estrogen receptor degrader) versus standard endocrine therapy for estrogen receptor-positive, human epidermal growth factor receptor 2-negative advanced breast cancer: results from the randomized phase III EMERALD trial. J Clin Oncol.

[44] Oliveira M, Pominchuck D, Nowecki Z (2023). Abstract GS3-02: GS3-02 camizestrant, a next generation oral SERD vs fulvestrant in post-menopausal women with advanced ER-positive HER2-negative breast cancer: results of the randomized, multi-dose phase 2 SERENA-2 trial. Cancer Research.

[45] Martin Jimenez M, Lim E, Chavez Mac Gregor M (2022). 211MO giredestrant (GDC-9545) vs physician choice of endocrine monotherapy (PCET) in patients (pts) with ER+, HER2- locally advanced/metastatic breast cancer (LA/mBC): primary analysis of the phase II, randomised, open-label acelERA BC study. Ann Oncol.

[46] Hamilton E, Vahdat L, Han HS (2022). Abstract PD13-08: first-in-human safety and activity of ARV-471, a novel PROTAC® estrogen receptor degrader, in ER+/HER2- locally advanced or metastatic breast cancer. Cancer Research.

[47] Dustin D, Gu G, Fuqua SAW (2019). ESR1 mutations in breast cancer. Cancer.

[48] Turner N, Huang-Bartlett C, Kalinsky K (2023). Design of SERENA-6, a phase III switching trial of camizestrant in *ESR1*-mutant breast cancer during first-line treatment. Future Oncol.

[49] Bhave MA, Quintanilha JCF, Tukachinsky H (2024). Comprehensive genomic profiling of ESR1, PIK3CA, AKT1, and PTEN in HR(+)HER2(-) metastatic breast cancer: prevalence along treatment course and predictive value for endocrine therapy resistance in real-world practice. Breast Cancer Res Treat.

[51] Chaudhary N, Chibly AM, Collier A (2024). CDK4/6i-treated HR+/HER2- breast cancer tumors show higher ESR1 mutation prevalence and more altered genomic landscape. NPJ Breast Cancer.

[52] Lloyd MR, Brett JO, Carmeli A et al (2024). CDK4/6 inhibitor efficacy in *ESR1*-mutant metastatic breast cancer. NEJM Evidence.

[53] Jhaveri K, Eli LD, Wildiers H (2023). Neratinib + fulvestrant + trastuzumab for HR-positive, HER2-negative, HER2-mutant metastatic breast cancer: outcomes and biomarker analysis from the SUMMIT trial. Ann Oncol.

[54] Desmedt C, Pingitore J, Rothé F (2019). ESR1 mutations in metastatic lobular breast cancer patients. NPJ Breast Cancer.

[55] Modi S, Jacot W, Yamashita T et al (2022). Trastuzumab deruxtecan in previously treated HER2-low advanced breast cancer. N Engl J Med.

[56] Almstedt K, Krauthauser L, Kappenberg F (2023). Discordance of HER2-low between primary tumors and matched distant metastases in breast cancer. Cancers.

[57] Miglietta F, Griguolo G, Bottosso M (2021). Evolution of HER2-low expression from primary to recurrent breast cancer. NPJ Breast Cancer.

[58] Curigliano G, Hu X, Dent RA (2024). Trastuzumab deruxtecan (T-DXd) vs physician’s choice of chemotherapy (TPC) in patients (pts) with hormone receptor-positive (HR+), human epidermal growth factor receptor 2 (HER2)-low or HER2-ultralow metastatic breast cancer (mBC) with prior endocrine therapy (ET): primary results from DESTINY-Breast06 (DB-06). JCO.

[59] Bardia A, Hu X, Dent R et al (2024). Trastuzumab deruxtecan after endocrine therapy in metastatic breast cancer. N Engl J Med.

[60] Litton JK, Rugo HS, Ettl J (2018). Talazoparib in patients with advanced breast cancer and a germline BRCA mutation. N Engl J Med.

[61] Robson M, Im SA, Senkus E (2017). Olaparib for metastatic breast cancer in patients with a germline BRCA mutation. N Engl J Med.

[62] Robson ME, Im SA, Senkus E (2023). OlympiAD extended follow-up for overall survival and safety: olaparib versus chemotherapy treatment of physician’s choice in patients with a germline BRCA mutation and HER2-negative metastatic breast cancer. Eur J Cancer.

[63] Robson ME, Tung N, Conte P (2019). OlympiAD final overall survival and tolerability results: olaparib versus chemotherapy treatment of physician's choice in patients with a germline BRCA mutation and HER2-negative metastatic breast cancer. Ann Oncol.

[64] Tung NM, Robson ME, Ventz S (2020). TBCRC 048: phase II study of olaparib for metastatic breast cancer and mutations in homologous recombination-related genes. J Clin Oncol.

[65] Condorelli R, Spring L, O'Shaughnessy J (2018). Polyclonal RB1 mutations and acquired resistance to CDK 4/6 inhibitors in patients with metastatic breast cancer. Ann Oncol.

[66] André F, Su F, Solovieff N (2023). Pooled ctDNA analysis of MONALEESA phase III advanced breast cancer trials. Ann Oncol.

[67] Gerratana L, Davis AA, Velimirovic M (2023). Cyclin-dependent kinase 4/6 inhibitors beyond progression in metastatic breast cancer: a retrospective real-world biomarker analysis. JCO Precis Oncol.

[68] Davis AA, Luo J, Zheng T (2023). Genomic complexity predicts resistance to endocrine therapy and CDK4/6 inhibition in hormone receptor-positive (HR+)/HER2-negative metastatic breast cancer. Clin Cancer Res.

[69] https://www.cancer.gov/tcga.

[70] AACR Project GENIE Consortium, André F, Arnedos M et al (2017). AACR project GENIE: powering precision medicine through an international consortium. Cancer Discov.

[71] Abu-Khalaf M, Wang C, Zhang Z (2022). Genomic aberrations in circulating tumor DNAs from palbociclib-treated metastatic breast cancer patients reveal a novel resistance mechanism. Cancers.

[72] Wander SA, Cohen O, Gong X (2020). The genomic landscape of intrinsic and acquired resistance to cyclin-dependent kinase 4/6 inhibitors in patients with hormone receptor-positive metastatic breast cancer. Cancer Discov.

[73] Al-Qasem AJ, Alves CL, Ehmsen S (2022). Co-targeting CDK2 and CDK4/6 overcomes resistance to aromatase and CDK4/6 inhibitors in ER+ breast cancer. NPJ Precis Oncol.

[74] Guarducci C, Bonechi M, Benelli M (2018). Cyclin E1 and Rb modulation as common events at time of resistance to palbociclib in hormone receptor-positive breast cancer. NPJ Breast Cancer.

[75] Caruso JA, Duong MT, Carey JPW, Hunt KK, Keyomarsi K (2018). Low-molecular-weight cyclin E in human cancer: cellular consequences and opportunities for targeted therapies. Cancer Res.

[76] Karakas C, Biernacka A, Bui T (2016). Cytoplasmic cyclin E and phospho-cyclin-dependent kinase 2 are biomarkers of aggressive breast cancer. Am J Pathol.

[77] Pascual J, Gil-Gil M, Proszek P (2023). Baseline mutations and ctDNA dynamics as prognostic and predictive factors in ER-positive/HER2-negative metastatic breast cancer patients. Clin Cancer Res.

[78] O'Leary B, Cutts RJ, Huang X (2021). Circulating tumor DNA markers for early progression on fulvestrant with or without palbociclib in ER+ advanced breast cancer. J Natl Cancer Inst.

[79] Cardoso F, Paluch-Shimon S, Senkus E (2020). 5th ESO-ESMO international consensus guidelines for advanced breast cancer (ABC 5). Ann Oncol.

[80] Lambertini M, Blondeaux E, Bisagni G (2023). Prognostic and clinical impact of the endocrine resistance/sensitivity classification according to international consensus guidelines for advanced breast cancer: an individual patient-level analysis from the Mammella InterGruppo (MIG) and Gruppo Italiano Mammella (GIM) studies. EClinicalMedicine.

[81] Agostinetto E, Sotiriou C, Ignatiadis M (2023). Clinico-molecular characteristics associated with outcomes in breast cancer patients treated with CDK4/6 inhibitors: results from the AURORA molecular screening initiative. JCO.

[82] Guerrero A, Alvarez I, Antolín Novoa S (2024). Comprehensive clinical characteristics and ctDNA mutational profile analysis of endocrine resistance/sensitivity to adjuvant ET therapy in luminal breast cancer from the GEICAM/2014-03_RegistEM registry. JCO.

[83] Llombart-Cussac A, Pérez-García JM, Bellet- Ezquerra M et al https://de170d6b23836ee9498a-9e3cbe05dc55738dcbe22366a8963ae7.ssl.cf1.rackcdn.com/2564236-1791205-003.pdf.

[84] (2012). Genome Atlas Network. Comprehensive molecular portraits of human breast tumours. Nature.

[85] Kalinsky K, Jacks LM, Heguy A (2009). PIK3CA mutation associates with improved outcome in breast cancer. Clin Cancer Res.

[86] Hortobagyi GN, Stemmer SM, Burris HA (2018). Updated results from MONALEESA-2, a phase III trial of first-line ribociclib plus letrozole versus placebo plus letrozole in hormone receptor-positive, HER2-negative advanced breast cancer. Ann Oncol.

[87] Tolaney SM, Toi M, Neven P (2022). Clinical significance of PIK3CA and ESR1 mutations in circulating tumor DNA: analysis from the MONARCH 2 study of abemaciclib plus fulvestrant. Clin Cancer Res.

[88] Baselga J, Im SA, Iwata H (2017). Buparlisib plus fulvestrant versus placebo plus fulvestrant in postmenopausal, hormone receptor-positive, HER2-negative, advanced breast cancer (BELLE-2): a randomised, double-blind, placebo-controlled, phase 3 trial. Lancet Oncol.

[89] Rugo HS, Lerebours F, Ciruelos E (2021). Alpelisib plus fulvestrant in PIK3CA-mutated, hormone receptor-positive advanced breast cancer after a CDK4/6 inhibitor (BYLieve): one cohort of a phase 2, multicentre, open-label, non-comparative study. Lancet Oncol.

[90] Bello Roufai D, Gonçalves A, De La Motte Rouge T (2023). Alpelisib and fulvestrant in PIK3CA-mutated hormone receptor-positive HER2-negative advanced breast cancer included in the French early access program. Oncogene.

[91] Turner S, Chia S, Kanakamedala H (2021). Effectiveness of alpelisib + Fulvestrant compared with real-world standard treatment among patients with HR+, HER2-, PIK3CA-mutated breast cancer. Oncologist.

[92] Jones RH, Casbard A, Carucci M (2020). Fulvestrant plus capivasertib versus placebo after relapse or progression on an aromatase inhibitor in metastatic, oestrogen receptor-positive breast cancer (FAKTION): a multicentre, randomised, controlled, phase 2 trial. Lancet Oncol.

[94] Howell SJ, Casbard A, Carucci M (2022). Fulvestrant plus capivasertib versus placebo after relapse or progression on an aromatase inhibitor in metastatic, oestrogen receptor-positive, HER2-negative breast cancer (FAKTION): overall survival, updated progression-free survival, and expanded biomarker analysis from a randomised, phase 2 trial. Lancet Oncol.

[95] Oliveira M, Rugo H, Howell S (2023). 187O capivasertib and fulvestrant for patients (pts) with aromatase inhibitor (AI)-resistant HR+/HER2- advanced breast cancer (ABC): subgroup analyses from the phase III CAPItello-291 trial. ESMO Open.

[96] Herrera-Abreu MT, Palafox M, Asghar U (2016). Early adaptation and acquired resistance to CDK4/6 inhibition in estrogen receptor-positive breast cancer. Cancer Res.

[97] Pascual J, Lim JSJ, Macpherson IR (2021). Triplet therapy with palbociclib, taselisib, and fulvestrant in PIK3CA-mutant breast cancer and doublet palbociclib and taselisib in pathway-mutant solid cancers. Cancer Discov.

[98] Tolaney SM, Im YH, Calvo E (2021). Phase Ib study of ribociclib plus fulvestrant and ribociclib plus fulvestrant plus PI3K inhibitor (alpelisib or buparlisib) for HR^+^ advanced breast cancer. Clin Cancer Res.

[99] Henry NL, Somerfield MR, Dayao Z (2022). Biomarkers for systemic therapy in metastatic breast cancer: ASCO Guideline Update. J Clin Oncol.

[100] Rugo HS, Raskina K, Schrock AB (2023). Biology and targetability of the extended spectrum of PIK3CA mutations detected in breast carcinoma. Clin Cancer Res.

[101] Schiavon G, Hrebien S, Garcia-Murillas I (2015). Analysis of ESR1 mutation in circulating tumor DNA demonstrates evolution during therapy for metastatic breast cancer. Sci Transl Med.

[102] Fribbens C, O'Leary B, Kilburn L (2016). Plasma ESR1 mutations and the treatment of estrogen receptor-positive advanced breast cancer. J Clin Oncol.

[103] Raghav KPS, Siena S, Takashima A (2023). Trastuzumab deruxtecan (T-DXd) in patients (pts) with HER2-overexpressing/amplified (HER2+) metastatic colorectal cancer (mCRC): Primary results from the multicenter, randomized, phase 2 DESTINY-CRC02 study. JCO.

[104] Bardia A, Bidard F, Neven P (2023). Abstract GS3-01: GS3-01 EMERALD phase 3 trial of elacestrant versus standard of care endocrine therapy in patients with ER+/HER2- metastatic breast cancer: updated results by duration of prior CDK4/6i in metastatic setting. Cancer Research.

[106] Tarantino P, Hamilton E, Tolaney SM (2020). HER2-low breast cancer: pathological and clinical landscape. J Clin Oncol.

[107] Molinelli C, Bruzzone M, Blondeaux E (2024). The journey of patients affected by metastatic hormone receptor-positive/HER2-negative breast cancer from CDK 4/6 inhibitors to second-line treatment: a real-world analysis of 701 patients enrolled in the GIM14/BIOMETA study. Eur J Cancer.

[108] Vaz Batista M, Pérez-García JM, Cortez P (2024). Trastuzumab deruxtecan in patients with previously treated HER2-low advanced breast cancer and active brain metastases: the DEBBRAH trial. ESMO Open.

[109] Zou Y, Yang A, Chen B (2024). crVDAC3 alleviates ferroptosis by impeding HSPB1 ubiquitination and confers trastuzumab deruxtecan resistance in HER2-low breast cancer. Drug Resist Updat.

[110] Pereira B, Chin SF, Rueda OM (2016). The somatic mutation profiles of 2,433 breast cancers refines their genomic and transcriptomic landscapes. Nat Commun.

[112] Nayar U, Cohen O, Kapstad C (2019). Acquired HER2 mutations in ER^+^ metastatic breast cancer confer resistance to estrogen receptor-directed therapies. Nat Genet.

[113] Gudmundsdottir K, Ashworth A (2006). The roles of BRCA1 and BRCA2 and associated proteins in the maintenance of genomic stability. Oncogene.

[114] Bruno L, Ostinelli A, Waisberg F (2022). Cyclin-dependent kinase 4/6 inhibitor outcomes in patients with advanced breast cancer carrying germline pathogenic variants in DNA repair-related genes. JCO Precis Oncol.

[115] Safonov A, Bandlamudi C, de Lara PT (2022). Abstract GS4-08: comprehensive genomic profiling of patients with breast cancer identifies germline-somatic interactions mediating therapy resistance. Cancer Research.

[116] Park SY, Suh KJ, Lee DW (2022). Prognostic role of tumor subtype and germline BRCA mutation in advanced breast cancer patients treated with palbociclib plus endocrine therapy. Breast Cancer Res Treat.

[117] Lee S, Park K, Kim GM (2022). Exploratory analysis of biomarkers associated with clinical outcomes from the study of palbociclib plus endocrine therapy in premenopausal women with hormone receptor-positive, HER2-negative metastatic breast cancer. Breast.

[118] Litton JK, Hurvitz SA, Mina LA (2020). Talazoparib versus chemotherapy in patients with germline BRCA1/2-mutated HER2-negative advanced breast cancer: final overall survival results from the EMBRACA trial. Ann Oncol.

[119] Curigliano G, Castelo-Branco L, Gennari A, Harbeck N, Criscitiello CT https://www.esmo.org/living-guidelines/esmo-metastatic-breast-cancer-living-guideline.

[120] Han E, Yoo J, Chae H (2020). Detection of BRCA1/2 large genomic rearrangement including BRCA1 promoter-region deletions using next-generation sequencing. Clin Chim Acta.

[121] Cam H, Dynlacht BD (2003). Emerging roles for E2F: beyond the G1/S transition and DNA replication. Cancer Cell.

[122] Li Z, Zou W, Zhang J (2020). Mechanisms of CDK4/6 inhibitor resistance in luminal breast cancer. Front Pharmacol.

[123] Mayer EL, Ren Y, Wagle N (2024). PACE: a randomized phase II study of fulvestrant, palbociclib, and avelumab after progression on cyclin-dependent kinase 4/6 inhibitor and aromatase inhibitor for hormone receptor-positive/human epidermal growth factor receptor-negative metastatic breast cancer. J Clin Oncol.

[124] Pascual J, Attard G, Bidard FC (2022). ESMO recommendations on the use of circulating tumour DNA assays for patients with cancer: a report from the ESMO precision medicine working group. Ann Oncol.

[125] Albanell J, Pérez-García JM, Gil-Gil M (2023). Palbociclib rechallenge for hormone receptor-positive/HER-negative advanced breast cancer: findings from the phase II BioPER Trial. Clin Cancer Res.

[126] Malorni L, Piazza S, Ciani Y (2016). A gene expression signature of retinoblastoma loss-of-function is a predictive biomarker of resistance to palbociclib in breast cancer cell lines and is prognostic in patients with ER positive early breast cancer. Oncotarget.

[127] Gong X, Litchfield LM, Webster Y (2017). Genomic aberrations that activate D-type cyclins are associated with enhanced sensitivity to the CDK4 and CDK6 inhibitor abemaciclib. Cancer Cell.

[128] Finn RS, Crown JP, Lang I (2015). The cyclin-dependent kinase 4/6 inhibitor palbociclib in combination with letrozole versus letrozole alone as first-line treatment of oestrogen receptor-positive, HER2-negative, advanced breast cancer (PALOMA-1/TRIO-18): a randomised phase 2 study. Lancet Oncol.

[129] Yang C, Li Z, Bhatt T (2017). Acquired CDK6 amplification promotes breast cancer resistance to CDK4/6 inhibitors and loss of ER signaling and dependence. Oncogene.

[130] Formisano L, Lu Y, Servetto A (2019). Aberrant FGFR signaling mediates resistance to CDK4/6 inhibitors in ER+ breast cancer. Nat Commun.

[131] Fagundes R, Teixeira LK (2021). Cyclin E/CDK2: DNA replication, replication stress and genomic instability. Front Cell Dev Biol.

[132] Keyomarsi K, Tucker SL, Buchholz TA (2002). Cyclin E and survival in patients with breast cancer. N Engl J Med.

[133] Wander SA, Han HS, Zangardi ML (2021). Clinical outcomes with abemaciclib after prior CDK4/6 inhibitor progression in breast cancer: a multicenter experience. J Natl Compr Canc Netw.

[134] Vijayaraghavan S, Karakas C, Doostan I (2017). CDK4/6 and autophagy inhibitors synergistically induce senescence in Rb positive cytoplasmic cyclin E negative cancers. Nat Commun.

[135] Pascual J, Gil-gil M, Zielinski C (2021). CCNE1 mRNA and cyclin E1 protein expression as predictive biomarkers for efficacy of palbociclib plus fulvestrant versus capecitabine in the phase III PEARL study. JCO.

[136] Guerrero A, Gil Gil MJ, Zielinski C (2023). Cyclin E cytoplasmatic isoform to predict outcome and benefit to capecitabine treatment in patients with HR+/HER2- metastatic breast cancer from the GEICAM/2013-02 PEARL study. JCO.

[137] Knudsen ES, Kumarasamy V, Nambiar R (2022). CDK/cyclin dependencies define extreme cancer cell-cycle heterogeneity and collateral vulnerabilities. Cell Rep.

[138] Zhang Z, Golomb L, Meyerson M (2022). Functional genomic analysis of CDK4 and CDK6 gene dependency across human cancer cell lines. Cancer Res.

[139] Grote I, Bartels S, Kandt L (2021). *TP53* mutations are associated with primary endocrine resistance in luminal early breast cancer. Cancer Med.

[140] Finn RS, Martin M, Rugo HS (2016). Palbociclib and letrozole in advanced breast cancer. N Engl J Med.

[141] Tripathy D, Im SA, Colleoni M (2018). Ribociclib plus endocrine therapy for premenopausal women with hormone-receptor-positive, advanced breast cancer (MONALEESA-7): a randomised phase 3 trial. Lancet Oncol.

[142] Hortobagyi GN, Stemmer SM, Burris HA (2022). Overall survival with ribociclib plus letrozole in advanced breast cancer. N Engl J Med.

[143] Garcia-Saenz JA, Blancas I, Echavarria I (2023). SEOM-GEICAM-SOLTI clinical guidelines in advanced breast cancer (2022). Clin Transl Oncol.

[144] Llombart-cussac A, Harper-wynne C, Perello A (2023). Second-line endocrine therapy (ET) with or without palbociclib (P) maintenance in patients (pts) with hormone receptor-positive (HR[+])/human epidermal growth factor receptor 2-negative (HER2[-]) advanced breast cancer (ABC): PALMIRA trial. JCO.

[145] Kalinsky K, Accordino MK, Chiuzan C (2023). Randomized phase II trial of endocrine therapy with or without ribociclib after progression on cyclin-dependent kinase 4/6 inhibition in hormone receptor-positive, human epidermal growth factor receptor 2-negative metastatic breast cancer: MAINTAIN trial. J Clin Oncol.

[146] Seki H, Sakurai T, Sakurada A, Kinoshita T, Shimizu K (2022). Subsequent-abemaciclib treatment after disease progression on palbociclib in patients with ER-positive HER2-negative metastatic breast cancer. Anticancer Res.

[147] Navarro-Yepes J, Kettner NM, Rao X (2023). Abemaciclib is effective in palbociclib-resistant hormone receptor-positive metastatic breast cancers. Cancer Res.

[148] Brett JO, Dubash TD, Johnson GN (2023). A gene panel associated with abemaciclib utility in *ESR1*-mutated breast cancer after prior cyclin-dependent kinase 4/6-inhibitor progression. JCO Precis Oncol.

[149] Kalinsky K, Bianchini G, Hamilton EP (2024). Abemaciclib plus fulvestrant vs fulvestrant alone for HR+, HER2- advanced breast cancer following progression on a prior CDK4/6 inhibitor plus endocrine therapy: primary outcome of the phase 3 postMONARCH trial. JCO.

[150] Schiff R, Jeselsohn R (2018). Is ctDNA the road map to the landscape of the clonal mutational evolution in drug resistance?. Cancer Discov.

[151] Baselga J, Campone M, Piccart M (2012). Everolimus in postmenopausal hormone-receptor-positive advanced breast cancer. N Engl J Med.

[152] Cook MM, Al Rabadi L, Kaempf AJ, Saraceni MM, Savin MA, Mitri ZI (2021). Everolimus plus exemestane treatment in patients with metastatic hormone receptor-positive breast cancer previously treated with CDK4/6 inhibitor therapy. Oncologist.

[153] Raphael A, Salmon-Divon M, Epstein J, Zahavi T, Sonnenblick A, Shachar SS (2022). Alpelisib efficacy in hormone receptor-positive HER2-negative PIK3CA-mutant advanced breast cancer post-everolimus treatment. Genes.

[154] Bardia A, Mayer IA, Vahdat LT (2019). Sacituzumab govitecan-hziy in refractory metastatic triple-negative breast cancer. N Engl J Med.

[155] Rugo HS, Bardia A, Marmé F (2022). Primary results from TROPiCS-02: a randomized phase 3 study of sacituzumab govitecan (SG) versus treatment of physician’s choice (TPC) in patients (Pts) with hormone receptor–positive/HER2-negative (HR+/HER2-) advanced breast cancer. JCO.

[156] Bardia A, Tolaney SM, Punie K (2021). Biomarker analyses in the phase III ASCENT study of sacituzumab govitecan versus chemotherapy in patients with metastatic triple-negative breast cancer. Ann Oncol.

[157] Mateo J, Chakravarty D, Dienstmann R (2018). A framework to rank genomic alterations as targets for cancer precision medicine: the ESMO Scale for Clinical Actionability of molecular Targets (ESCAT). Ann Oncol.

[158] Terraf P, Pareja F, Brown DN (2022). Comprehensive assessment of germline pathogenic variant detection in tumor-only sequencing. Ann Oncol.

[159] Chakravarty D, Johnson A, Sklar J (2022). Somatic genomic testing in patients with metastatic or advanced cancer: ASCO provisional clinical opinion. J Clin Oncol.

[160] Fisher RP (2019). Cdk7: a kinase at the core of transcription and in the crosshairs of cancer drug discovery. Transcription.

[161] Martin L, Pancholi S, Ribas R (2017). Abstract P3-03-09: resistance to palbociclib depends on multiple targetable mechanisms highlighting the potential of drug holidays and drug switching to improve therapeutic outcome. Cancer Research.

[162] Li B, Ni Chonghaile T, Fan Y (2017). Therapeutic rationale to target highly expressed CDK7 conferring poor outcomes in triple-negative breast cancer. Cancer Res.

[163] Sava GP, Fan H, Coombes RC, Buluwela L, Ali S (2020). CDK7 inhibitors as anticancer drugs. Cancer Metastasis Rev.

[164] Coombes RC, Howell S, Lord SR (2023). Dose escalation and expansion cohorts in patients with advanced breast cancer in a phase I study of the CDK7-inhibitor samuraciclib. Nat Commun.

[165] Stewart MD, Merino Vega D, Arend RC (2022). Homologous recombination deficiency: concepts, definitions, and assays. Oncologist.

[166] Llop-guevara A, Vladimirova V, Schneeweiss A (2021). 2O association of RAD51 with homologous recombination deficiency (HRD) and clinical outcomes in untreated triple-negative breast cancer (TNBC): analysis of the GeparSixto randomized clinical trial. Ann Oncol.

[167] Serra Elizalde V, Llop-guevara A, Pearson A (2021). 1O detection of homologous recombination repair deficiency (HRD) in treatment-naive early triple-negative breast cancer (TNBC) by RAD51 foci and comparison with DNA-based tests. Ann Oncol.

[168] Pack LR, Daigh LH, Chung M, Meyer T (2021). Clinical CDK4/6 inhibitors induce selective and immediate dissociation of p21 from cyclin D-CDK4 to inhibit CDK2. Nat Commun.

[169] Yap TA, Giordano A, Hamilton EP (2023). First-in-human first-in-class phase 1/2a study of the next generation CDK4-selective inhibitor PF-07220060 in patients (pts) with advanced solid tumors, enriched for HR+ HER2- mBC who progressed on prior CDK4/6 inhibitors and endocrine therapy. JCO.

[170] Yap TA, Elhaddad AM, Grisham RN (2023). First-in-human phase 1/2a study of a potent and novel CDK2-selective inhibitor PF-07104091 in patients (pts) with advanced solid tumors, enriched for CDK4/6 inhibitor resistant HR+/HER2- breast cancer. JCO.

[171] Arora M, Moser J, Hoffman TE (2023). Rapid adaptation to CDK2 inhibition exposes intrinsic cell-cycle plasticity. Cell.

[172] Dietrich C, Trub A, Ahn A (2024). INX-315, a selective CDK2 inhibitor, induces cell cycle arrest and senescence in solid tumors. Cancer Discov.

[173] Mukohara T, Park YH, Sommerhalder D (2024). A phase 1 dose expansion study of a first-in-class KAT6 inhibitor (PF-07248144) in patients with advanced or metastatic ER+ HER2- breast cancer. JCO.

[174] Jiang B, Bao L, He S, Chen X, Jin Z, Ye Y (2024). Deep learning applications in breast cancer histopathological imaging: diagnosis, treatment, and prognosis. Breast Cancer Res.

[175] Gamble P, Jaroensri R, Wang H (2021). Determining breast cancer biomarker status and associated morphological features using deep learning. Commun Med.

[176] Rakhshaninejad M, Fathian M, Shirkoohi R, Barzinpour F, Gandomi AH (2024). Refining breast cancer biomarker discovery and drug targeting through an advanced data-driven approach. BMC Bioinformatics.

[177] Moiso E, Ferraro E, Cabel L (2024). Predicting CDK4/6 inhibitors outcomes in pts with HR+/HER2- metastatic breast cancer: a machine learning approach. JCO.

