# Supplementary material for: Optimizing therapeutic approaches for HR+/HER2- advanced breast cancer: clinical perspectives on biomarkers and treatment strategies post-CDK4/6 inhibitor progression
Source: Cancer Drug Resist. 2025 Jan 22;8:5. doi: 10.20517/cdr.2024.169 (PMC11810462; doi:10.20517/cdr.2024.169)
Supplement: Supplementary file 1 [file cdr-8-5-SupplementaryMaterials.pdf]

Supplementary Materials

Optimizing therapeutic approaches for HR+/HER2- advanced breast cancer: clinical perspectives on biomarkers and treatment strategies post-CDK4/6 inhibitor progression

Juan Miguel Cejalvo Andújar[1,2,3], Francisco Ayala de la Peña[4], Mireia Margeli Vila[5,6], Javier Pascual[3,7], Pablo Tolosa[8], Cristina Pages[9], Mónica Cuenca[9], Ángel Guerrero Zotano[10]

[1]Medical Oncology Department, Hospital Clínico Universitario de Valencia, Valencia 46010, Spain.

[2]INCLIVA Biomedical Research Institute, Valencia 46010, Spain.

[3]Center for Biomedical Network Research on Cancer (CIBERONC), Madrid 28019, Spain.

[4]Medical Oncology Department, Hospital General Universitario Morales Meseguer, Murcia 30008, Spain.

[5]Medical Oncology Department, Instituto Catalán de Oncología, Badalona 08916, Spain.

[6]CARE, the Translational Program in Cancer Research of Germans Trias i Pujol Research Institute (IGTP), Badalona 08916, Spain.

[7]Medical Oncology Department, UGC Intercentros de Oncología Médica, Hospitales Universitarios Regional y Virgen de la Victoria, IBIMA, Málaga 29010, Spain.

[8]Medical Oncology Department, Hospital Universitario 12 de octubre, Madrid 28041, Spain.

[9]Medical Department, Pfizer Oncology, Madrid 28108, Spain.

[10]Medical Oncology Department, Instituto Valenciano de Oncología, Valencia 46009, Spain.

Correspondence to: Dr. Ángel Guerrero Zotano, Medical Oncology Department, Instituto Valenciano de Oncología, Carrer del Professor Beltrán Báguena 8, Campanar, Valencia 46009, Spain. E-mail: angel.guerrero.zotano@gmail.com

Supplementary Table 1. Overview of main umbrella trials investigating second-line treatments in HR+/HER2- advanced breast cancer (ABC) following progression on CDK4/6 inhibitors combined with endocrine therapy (ET)

Trial name (Trial ID)	Phase	Treatment investigated	Estimated completion date	Termination Status
**ELEVATE (NCT05563220)**	Phase Ib/II	Elacestrant/alpelisib, everolimus, abemaciclib/ribociclib, palbociclib/abemaciclib/ribociclib, capivasertib	2026-08	Recruiting
**MULAN (NCT04355858)**	Phase II	SHR7390, famitinib, SHR3162, pyrotinib, capecitabine, SHR1210, everolimus, nab paclitaxel, SHR2554, SHR3680, SHR6390, SHR1701, fulvestrant, aromatase inhibitor, bevacizumab	2025-04	Recruiting
**TACTIVE-U (sub-study A: NCT05548127; sub-study B: NCT05573555)**	Phase Ib/II	ARV-471/abemaciclib (sub-study A); ARV-471/ribociclib (sub-study B)	2025-09 (sub-study A) 2027-01 (sub-study B)	Active, not recruiting (sub-study A) Recruiting (sub-study B)
**MORPHEUS- BREAST CANCER (NCT04802759)**	Phase Ib/II	Giredestrant monotherapy or giredestrant/abemaciclib, ipatasertib, inavolisib, ribociclib, everolimus, samuraciclib, atezolizumab, abemaciclib/atezolizumab (Cohort 1)	2026-10	Recruiting
**Morpheus-panBC (NCT03424005)**	Phase I/II	Inavolisib/abemaciclib/fulvestrant, inavolisib/ribociclib/fulvestrant, inavolisib/ribociclib/letrozole, inavolisib/abemaciclib/letrozole (Cohort 3)	2028-05	Recruiting
**MORPHEUS-HR+ Breast Cancer** [1] **(NCT03280563)**	Phase I/II	Fulvestrant (control), fulvestrant/atezolizumab, fulvestrant/atezolizumab/abemaciclib	2024-09	Completed

**OP-1250-003 (NCT05508906)**	Phase I	OP-1250 (palazestrant)/ribociclib, alpelisib, everolimus	2026-06	Recruiting
**GERTRUDE (NCT05933395)**	Phase II	Neratinib/fulvestrant, alpelisib/fulvestrant, everolimus/fulvestrant, abemaciclib/fulvestrant	2031-10	Recruiting
**ELAINE-3 (NCT05696626)**	Phase III	Lasofoxifene/abemaciclib, fulvestrant/abemaciclib	2028-04	Recruiting

BC: Breast cancer; HR: hormone receptor.

Cited reference: 1. Jung KH, Im S-A, Yardley D et al. Abstract PS12-08: MORPHEUS Hormone Receptor-Positive Breast Cancer: interim analysis of a Phase Ib/II, study of fulvestrant ± atezolizumab and abemaciclib triplet treatment in patients metastatic disease. Cancer Research 2024;84:PS12-08-PS12-08. [DOI: 10.1158/1538-7445.SABCS23-PS12-08]
